# Exacerbation of Mania due to Metronidazole in a Bipolar Disorder Patient

**DOI:** 10.1155/2022/3748101

**Published:** 2022-06-02

**Authors:** Majed AlShakori, Savera I. Arain, Shabeer A. Thorakkattil, Syed Abdulkader

**Affiliations:** ^1^Pharmacy Services Department, Johns Hopkins Aramco Healthcare (JHAH), Saudi Arabia; ^2^Behavioral Health Clinic, Johns Hopkins Aramco Healthcare (JHAH), Saudi Arabia

## Abstract

Bipolar disorder is a mental health disorder where the patient experiences extreme shifts in mood marked by depression, mania, or hypomania. It affects their overall daily life activities and sleep patterns. This case report is of a 74-year-old female patient with bipolar disorder who experienced a manic episode after initiation of antibiotics to treat gallbladder perforation with abscess formation. The patient's past medical history included Parkinson's disease, diabetes mellitus, bipolar disorder, and acalculous cholecystitis. The patient required hospitalization for a cholecystostomy tube insertion for drainage. During hospitalization, the patient was started on empiric treatment with broad-spectrum antibiotics, including piperacillin/tazobactam and metronidazole. The patient remained stable during the inpatient stay and was discharged home one week later. She was prescribed cefuroxime and metronidazole to complete a 2-week duration of antibiotics. However, upon discharge, she developed manic symptoms, including lack of need to sleep, excessive talking, and severe agitation. Upon assessment, the psychiatric team decided to hold metronidazole as it has an adverse effect of mania as evidenced in drug information resources. The patient started to show immediate recovery from the symptoms with complete resolution of manic symptoms on the 3^rd^ day following the discontinuation of metronidazole. This case emphasizes the increased need for vigilance in bipolar patients upon prescribing metronidazole. Also, further research is needed to predict the time to onset of manic symptoms and improvement in patient symptoms upon drug discontinuation.

## 1. Introduction

Bipolar disorder is a mental disorder that causes changes in a person's emotional status affecting their mood and energy. It causes a lack of interest in daily activities and affects the patients' work, social interactions, and overall performance. People with bipolar disorder often experience either manic, hypomanic, or depressive mood episodes. The manic episode is marked by symptoms including a feeling of high, elated and restlessness, loss of appetite, racing thoughts, and decreased need to sleep. The depressive episode includes feeling unhappy or empty, decreased appetite, loss of pleasure in daily activities, social isolation, and suicidal thoughts. Given the rise in the geriatric population, there are an increased number of geriatric patients with bipolar disorder. It is particularly challenging to treat bipolar disorder in this patient population as they have comorbid conditions and are more sensitive to adverse reactions from medications [1–3].

Geriatric patients are more susceptible to adverse reactions from medications due to the presence of comorbidities, multiple medications, and both pharmacokinetics and pharmacodynamics age-related changes. Both drug-drug and drug-disease interactions need to be carefully monitored [4]. Several incidents of neurotoxic adverse effects have been reported in geriatric patients who have been on antimicrobial medications. The neurotoxic adverse effect may easily be missed in this patient population, which makes it crucial to prescribe antimicrobials carefully and monitor any new onset of side effects or disease-related complications [5, 6].

## 2. Case Presentation

The patient is a 74-year-old female with a previous medical history significant of multiple medical comorbidities, including Parkinson's disease, diabetes mellitus, bipolar disorder, and acalculous cholecystitis. The patient presented to the emergency department complaining of right upper quadrant pain for 1 week with episodes of fever. CT of the abdomen revealed a perforated gallbladder with the collection. The patient's admission plan included alleviating the infection and significant inflammatory process using antibiotics and percutaneous drainage. She underwent the procedure of cholecystostomy tube insertion for drainage, which was well tolerated. She reported well-controlled pain and slept well. All psychiatric consult visits showed that she remained composed and cooperative with no changes or fluctuations in her mood.

The patients prior to admission medications included aspirin 81 mg daily, atorvastatin 20 mg daily, carbidopa-levodopa 25-100 mg twice daily, lamotrigine 100 mg twice daily, insulin mixtard twice daily, mirtazapine 30 mg daily, risperidone 1 mg at bedtime, sodium valproate 500 mg daily, vitamin D3 (cholecalciferol) once monthly, and zolpidem 10 mg at bedtime.

During admission, patient received acetaminophen (as needed for pain), aspirin 81 mg daily, atorvastatin 20 mg daily, carbidopa-levodopa 25-100 twice daily, lamotrigine 100 mg twice daily, losartan-hydrochlorothiazide (50/12.5 mg) once daily, metronidazole 500 mg three times daily, miralax as needed for constipation, piperacillin/tazobactam 3.375 grams IV every 8 hours, mirtazapine 30 mg daily, omeprazole 20 mg daily, risperidone 1 mg at bedtime, sodium valproate 500 mg daily, and zolpidem 10 mg at bedtime.

On 7^th^ day of admission and postcholecystostomy tube insertion, there was no drainage reported post 5th day. The patient was otherwise stable, and after consultation with the radiologist, a cholecystostomy tube was removed and the patient was discharged home. Upon discharge, she received acetaminophen (as needed for pain), aspirin 81 mg daily, atorvastatin 20 mg daily, carbidopa-levodopa 25-100 twice daily, lamotrigine 100 mg twice daily, losartan-hydrochlorothiazide (50/12.5 mg) once daily, metronidazole 500 mg three times daily, cefuroxime 500 mg twice daily, mirtazapine 30 mg daily, risperidone 1 mg at bedtime, sodium valproate 500 mg daily, and zolpidem 10 mg at bedtime.

On the 2^nd^ day postdischarge, the patient was brought to the emergency room by her family. According to her son, the patient was experiencing an episode of significant confusion, severe insomnia, bilateral upper extremity tremors, and repetition of her words for almost one hour. She was given an anxiolytic and admitted for observation and further workup. Neurology, cardiology, and psychiatric consults were opened. Postworkup, all her labs returned normal except for slightly elevated troponin. Repeat troponin; however, it returned normal. The cardiologist pointed that sometimes cholecystitis can cause troponin elevation. The neurology consult was insignificant, with no evidence of seizures or tremors. The psychiatric consult noted that she was sleeping well after receiving lorazepam. Following an interdisciplinary round on that same day, the patient was discharged home again and resumed on the same home medication as upon previous discharge. On 2^nd^ day postdischarge, the patient's daughter contacted the psychiatric clinic. She reported that the patient showed a recent change in mental status, cycling between depressive symptoms (excessive crying) to mania (talkative and decreased sleep) suggestive of mixed state or dysphoric mania. Upon medication reconciliation, due to the possibility of metronidazole-induced mania, the psychiatric team recommended holding the metronidazole as a trial after consultation with the internal medicine. The patient had already been covered with another broad-spectrum antibiotic for cholecystitis. The family was advised to monitor the mental health of the patient and return to the emergency department if patient's condition worsened. Within 24 hours of removing metronidazole, the patient started to show signs of recovery with a complete resolution of symptoms of mania over the next 2-3 days. The patient continued to be closely monitored over next several days and weeks and showed no recurrence of the manic episode.

## 3. Discussion

This is the first descriptive case report of suspected metronidazole-induced mania in a geriatric patient with bipolar disorder. Metronidazole is a widely used antibiotic for treating bacterial and parasitic infections. It is also used in combination with other antibiotics and for surgical prophylaxis. Its oral form is rapidly absorbed and has good distribution to almost entire body [6, 7]. Gall stones can be one of the causes of acute cholecystitis, and it should be treated quickly with intravenous hydration, analgesia, and prophylactic broad-spectrum antibiotics. Antibiotic coverage usually includes a combination of second-generation cephalosporin with metronidazole. Percutaneous cholecystostomy is a minimally invasive procedure and considered a safe alternative for patients with comorbid conditions [8].

The prescribing information for metronidazole includes a warning for seeking emergency help if the patient develops any mental or mood changes, including confusion, difficulty in speech, or severe headache [9], while the exact mechanism of metronidazole-induced neurotoxicity remains unclear, and studies suggest damage to nerve fibers from the free radicals or the inhibition of protein synthesis and axonal degeneration of nerve fiber when metronidazole and its metabolites combined with RNA as possible causative mechanisms [7]. While the incidence of only 0.25% for developing neurotoxicity with symptoms including ataxia and confusion among patients is reported, it is most likely an underestimate. Also, the onset varies with some patients developing symptoms within a few days vs. others in 6-7 weeks. Neurotoxicity risk is also reported to be higher in patients with IBD, osteomyelitis, and undrained abscesses. In the majority of cases, patients showed complete recovery from neurotoxic symptoms upon discontinuation of medication within a few days of discontinuing metronidazole [10, 11].

This patient had a long history of bipolar disorder and had continued to remain stable on her current medications. Patient has not been prescribed metronidazole previously as reported by the family or noted in her electronic medical record. The psychiatric side effect of antibacterial is not a new phenomenon, and it has been previously reported with Iproniazid, an antitubercular drug which was recalled from the market due to excessive side effects [12–14].

Previously reported cases of antibiotic-induced mania (antibiomania) have found that there is a possible link between the use of antibiotics and psychiatric symptoms and disorders including schizophrenia and bipolar disorder. The antibiomania is more commonly seen with isoniazid, quinolones, cotrimoxazole, and macrolides, and suggestive mechanism includes a combination of the body's inflammatory response to infection and alteration of gut bacteria. In addition, gamma aminobutyric acid (GABA) is decreased from the use of these antibiotics. A systematic review shared hypothesis of quinolones competing for and inhibiting GABA receptor sites and clarithromycin antagonizing postsynaptic GABA-A receptors in neuron while isoniazid showed glutamic acid decarboxylase (GAD) enzyme inhibition [15–18]. Diagnostic and Statistical Manual of Mental Disorders, Fifth Edition (DSM-5) has listed antibiotic use as a causative factor for secondary mania [17]. Antibiotics are very commonly known to exert a modifying effect on the immune system in addition to exerting antimicrobial effect. Both psychotic and mood disorders are shown to have increased in patients' postdevelopment of a bacterial infection. While more studies are needed to find a conclusive mechanism causing the increased episodes of acute mania in psychiatric patients, some possible suggestions based on previous studies include activation of the immune system by the antibiotics, compromised immunity due to higher number of bacterial infections, and the antibiotic induced changes in gut microbiota [19]. One of the most important differential diagnostic tools is to observe the time of onset of the manic symptoms and the initiation of antimicrobial therapy. If a possible link exists, then discontinuation of the antibiotic is a crucial step. The number of antibiomania cases remains unknown for the most part due to lack of published studies, lack of calculative methods, and number of cases reported. Also, the link between the antibiotic and mania is peculiar as the studies have suggested that patients did not exhibit any previous occurrence of mental disorder, and the proinflammatory cytokines released in response to infection trigger the manic episodes [20].

More research is needed to establish the presence of manic episodes in bipolar disorder patients, especially in the geriatric population due to metronidazole. It is important for health care professionals and bipolar disorder patients to be aware of this rare adverse effect from the use of metronidazole in this particular patient population. Also, more studies are needed to explore time to onset, possible dose relationship, reversibility of this side effect upon discontinuation of the antibiotic, and its effect on subsequent use on the bipolar affective disorder.

## 4. Conclusion

Our case report shows that metronidazole can cause the development of manic episodes in bipolar disorder in geriatric patients. Close monitoring and increased awareness upon prescribing are therefore recommended in similar cases.

## Data Availability

The data will be available by contacting the corresponding using provided email.
